# Interventional Treatment for Portal Venous Occlusion After Liver Transplantation: Long-Term Follow-Up Results: Retraction

**DOI:** 10.1097/MD.0000000000050361

**Published:** 2026-08-14

**Authors:** Jianfeng Wang, Weili Yang, Qiang Huang, Kun Gao, Baojie Wei, Renyou Zhai, Yaoping Shi

The article, “Interventional Treatment for Portal Venous Occlusion After Liver Transplantation: Long-Term Follow-Up Results”,^[1]^ which appears in Volume 94, Issue 4 of *Medicine*, is being retracted due to insufficient evidence that the study did not use organs or data derived from executed prisoners. The authors were notified of this retraction.
